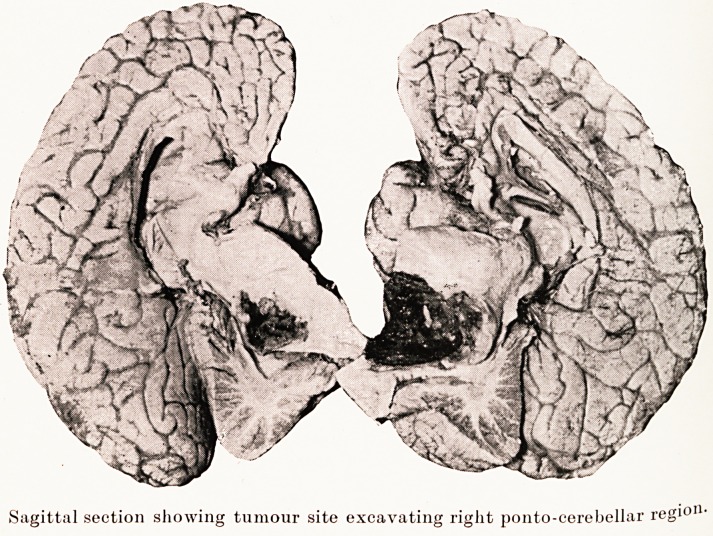# Note on a Case of Ponto-Cerebellar Tumour in a Girl of Six Years

**Published:** 1932

**Authors:** A. Wilfrid Adams

**Affiliations:** Assistant Surgeon, Bristol Royal Infirmary, and Surgeon, Bristol Royal Hospital for Sick Children and Women


### PLATE IV

Inferior aspect of right ponto-cerebellar region expanded by tumour from which 7th and 8th cranial nerves issue.

Sagittal section showing tumour site excavating right ponto-cerebellar region.

### NOTE ON A CASE OF PONTO-CEREBELLAR TUMOUR IN A GIRL OF SIX YEARS.

BY

A. Wilfrid Adams, M.S., F.R.C.S.,

*Assistant Surgeon, Bristol Royal Infirmary, and Surgeon, Bristol Royal Hospital for Sick Children and Women.*

---

J. W., female, æt. $8\frac{11}{12}$ years, was bright and healthy, but never strong. Whooping cough was the only definite trouble she had suffered till the beginning of February, 1932, when pain started behind the right ear and she held her head turned to the left. She was a patient of Dr. Walshman Ward. There was difficulty in micturition, constipation and irregular vomiting. This last ceased for a few weeks after admission into the Children's Hospital. There she was under the care of Dr. O. C. M. Davis, who kindly invited me to see her and operate.

She was jumpy when she walked into hospital, and for a few weeks her condition suggested chorea. Thereafter cerebral symptoms developed, the child lying quiet but conscious, and always preferring to turn her head to the left. Stupor set in, and vomiting returned in the last week. The tongue was furred, the temperature usually subnormal, and the pulse as often below as above 100. The abdomen was notably scaphoid.

In her last two weeks a left hemiplegia developed with slightly dilated pupil and increased reflexes on that side. Accompanying this there were impaired audition and facial palsy on the opposite side. The optic discs and fundi were found normal by Mr. Garden, and three examinations of cerebro-spinal fluid by Professor Walker Hall proved negative, including the Wassermann reaction. There were 36 mgs. of urea per 100 c.c. of blood, and the urine remained normal.

*Conclusions.*—Crossed paralysis present, due to right-sided tumour pressing on the right cranial nerves, and the left pyramidal tract above its decussation.

*Operation.*—On 18th April, 1932, an extensive right-sided decompression was performed, and obvious pulsation of the dura was noted. 10 c.c. of clear cerebro-spinal fluid (subsequent report normal) were aspirated from the right lateral ventricle. Her condition improved, but I did not feel further exploration warranted at the time, and the child died the following morning.

*Post-mortem.*—Revealed a cyst of the size of a pigeon's egg in the right cerebello-pontine region. The facial and auditory nerves appeared to run out of it, and as the pons was hollowed out markedly, it may perhaps best be designated a ponto-cerebellar tumour. It consisted mainly of dark greenish-grey semi-fluid contents. Apparently necrosis and hæmorrhage had occurred.

*Biopsy.*—The special rapid examination of the specimen showed the presence of old and recent hæmorrhages, associated with neoplastic changes known formerly as "Angiosarcoma."

### REMARKS.

It is interesting, quoting from Critchley,[1] to note that while brain tumours are relatively rare in childhood, about 50 per cent. are subtentorial in position, and pontine tumours characteristically do not produce papillœdema. Although such a tumour is not amenable to excision owing to its site in the brain stem, the contents could have been sucked out—as advocated by Sargent,[2] with possible relief of symptoms.

### REFERENCES.

[1] Critchley, *British Journal of Children's Diseases*, October–December, 1925, vol. xxii., p. 251 *et seq.*

[2] Sargent, *Bristol Med.-Chir. Jour.*, 1925, vol. xlii., p. 101 *et seq.*

## Figures and Tables

**Figure f1:**
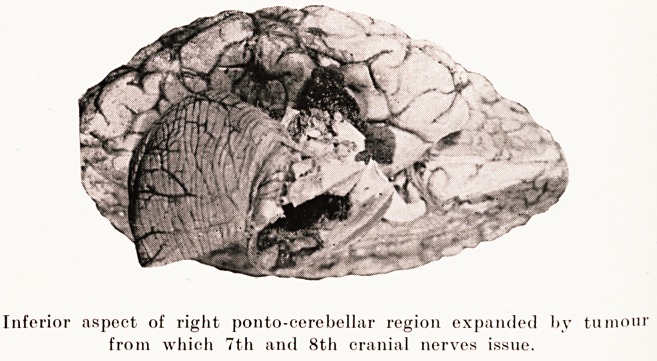


**Figure f2:**